# Calculation of Reaction Forces in the Boiler Supports Using the Method of Equivalent Stiffness of Membrane Wall

**DOI:** 10.1155/2014/392048

**Published:** 2014-05-15

**Authors:** Josip Sertić, Dražan Kozak, Ivan Samardžić

**Affiliations:** Mechanical Engineering Faculty in Slavonski Brod, Trg Ivane Brlić-Mažuranić 2, 35 000 Slavonski Brod, Croatia

## Abstract

The values of reaction forces in the boiler supports are the basis for the dimensioning of bearing steel structure of steam boiler. In this paper, the application of the method of equivalent stiffness of membrane wall is proposed for the calculation of reaction forces. The method of equalizing displacement, as the method of homogenization of membrane wall stiffness, was applied. On the example of “Milano” boiler, using the finite element method, the calculation of reactions in the supports for the real geometry discretized by the shell finite element was made. The second calculation was performed with the assumption of ideal stiffness of membrane walls and the third using the method of equivalent stiffness of membrane wall. In the third case, the membrane walls are approximated by the equivalent orthotropic plate. The approximation of membrane wall stiffness is achieved using the elasticity matrix of equivalent orthotropic plate at the level of finite element. The obtained results were compared, and the advantages of using the method of equivalent stiffness of membrane wall for the calculation of reactions in the boiler supports were emphasized.

## 1. Introduction

Nowadays, the steam boilers of waste incineration power plants are made specifically according to the requirements of investors. Due to the location where the boiler will be set up, the amount and type of fuel to be burned in a given time, and technical parameters of produced steam, the production of steam boilers is mainly individual. This represents an exceptional effort for the designers. The pipes and sheet metal are the basic structural elements used for making boilers. Larger structural units called the membrane walls are obtained by welding these elements. The membrane walls make the basic structure of boiler and have a double function. The heat exchanger, that is, evaporator, is the primary function, and the secondary is transfer of loading. The steam boiler membrane walls are loaded by the pressure of working fluid (water/steam), their own mass, and the mass of other structural units that rely on the membrane walls, the mass of working fluid, the mass of insulation and refractory, and the fouling mass that is deposited during the combustion process. The loading is transferred across the membrane walls to the boiler supports and then to the bearing steel structure.

Since the water tube boilers are large structures consisting of a large number of pipes connected to the membrane walls, it is very difficult to accurately calculate the reactions in the supports. In practice, there are two approaches to calculate the reactions in the steam boiler supports. The first approach is based on the assumption that the membrane stiffness of the boiler membrane wall is very high, so the boiler is calculated as an ideal rigid body. The second approach takes into account the finite stiffness of the membrane walls using the finite element method. The discretization of real geometry of boiler (membrane walls) requires an extremely large number of finite elements, so this approach is rarely used, eventually for smaller boilers. Most often, the membrane wall stiffness is approximated by the equivalent stiffness of orthotropic plate/shell, which allows a multiple reduction of degrees of freedom of a numerical model.

It is difficult to find more detailed research papers on the topic of homogenization of steam boiler membrane wall. In 2012, Milošević-Mitić [1] with a group of authors proposed a procedure for calculating the strength of water tube boiler using the finite element of orthotropic plate. The homogenization of membrane wall stiffness is performed numerically, using the method of equalizing displacement.

When calculating the deflection of membrane walls due to the influence of underpressure/overpressure of flue gases, the equivalent wall thickness method is most commonly used in practice. The bending stiffness of membrane wall pipe is equal to the bending stiffness of equivalent wall thickness for the axis perpendicular to the longitudinal axis of membrane wall pipe, and the bending stiffness of membrane wall tape is equal to the bending stiffness of equivalent wall thickness for the axis parallel to the longitudinal axis of membrane wall pipe.

The steam boiler membrane wall represents a structurally orthotropic plate. The stiffened plates that are frequently used, both in building and in the processing/power plants, where it is necessary to achieve the optimal structural solution considering the mass of structure, also belong to this class of structures. The earliest research on the topic of stiffness of stiffened plates was most likely done by Huber [2–4]. His investigations were inspired by the lack of exact expressions for solving the problems of bending and buckling of stiffened structures by using the theory of homogeneous orthotropic plates. Similarly, Flügge [5] published the first paper on the topic of structurally stiffened shell in 1932, and 15 years later, so did van der Neut [6]. Over time, the understanding of the theory of deformation of structurally orthotropic plates and shells has progressed. The terms “equivalent stiffness” and “equivalent wall thickness” became the basis for the analysis of deformation of stiffened or structurally orthotropic plates/shells. Furthermore, Dale et al. [7] applied the equivalent stiffness in the research of buckling of pressure loaded sandwich plates. Smith et al. [8] proposed an improved formulation that takes into consideration the change of position of the neutral surface that is connected with the local interaction between the plate and stiffener. Pflüger [9], Gomza and Seide [10], and Libove and Hubka [11] applied in their research the method of equivalent stiffness and thickness of stiffened plate/shell. In the papers [12–14], the theory of symmetric sandwich plates, with the included transverse shear stiffness, is applied. In 1956, Huffington Jr. [15] analyzed the equivalent stiffness of orthogonally stiffened plate without the stiffener eccentricity. During the last fifty years, the equivalent plate or shell approach was still being used as a first approximation method [16–19]. For the experimental determination of elasticity constants, the method of testing the load-deformation characteristics of repeating structural part of the structurally orthotropic plate was most commonly used [20]. In 1969, Soong [21–23] presented a derivation of the stiffness expressions for orthotropic cylinders based on a deformation energy approach. Throughout the following years, special attention was given to the interaction between the plate and stiffeners [24], for a different layout or grid of stiffeners [25–27] and different combinations of loads. In 1995, Jaunky et al. [28, 29] presented a refined smeared-stiffener theory for grid-stiffened laminated-composite panels, based upon the work presented by Smith et al. [8]. Similarly, Wodesenbet presented an improved smeared-stiffener theory for grid-stiffened laminated-composite cylinders in 2003.

It can be concluded that the current research is mainly based on two systematic analytical methods that take or do not take into account the transverse shear stiffness. First, there is a direct equilibrium-compatibility method. The first method uses equilibrium and compatibility in a direct manner for plates reinforced by stiffeners. The second one is called the basic-cell energy-equivalence method. The second method is based on defining the equivalence between the deformation energy of the basic repeating cell and the deformation energy of the equivalent orthotropic plate. This method is suitable for the determination of the members of elasticity matrix for grid-stiffened plates and sandwich plates.

Unlike the stiffened plates, a steam boiler membrane wall consists of a homogeneous plate with attached stiffeners. Therefore, the two mentioned methods are not entirely suitable for the determination of equivalent stiffness of membrane wall, that is, the equivalent elasticity constants. The process of homogenization of steam boiler membrane wall is presented in the papers [30–32]. The elasticity constants for the membrane and bending stiffness of membrane wall are determined theoretically, by equalizing the deformation of membrane wall with the deformation of equivalent orthotropic plate. In these papers, the transverse shear stiffness is not taken into consideration.

## 2. Steam Boiler Membrane Wall as the Structurally Orthotropic Plate

The steam boiler membrane wall can be approximated as the equivalent orthotropic plate, that is, the orthotropic plate, which will have the same elastic properties as the real membrane wall. Using Kirchhoff-Love theory of shells [33, 34], the constitutive equations of equivalent orthotropic plate or shell can be written as follows:

(1) $$\begin{array}{c} \sigma =D\cdot \varepsilon , \end{array}$$

where **σ** is vector of section forces, **D** is matrix of elasticity, and **ε** is vector of deformation. According to [35], it can be said that the constitutive equations for the analysis of plates and shells are analog. The matrix expression (1) represents the six constituent equations of membrane wall as the structurally orthotropic plate or its equivalent orthotropic plate, which connect the section forces with the corresponding deformations

(2) $$\begin{array}{c} \left[\begin{array}{c} N_{1}\\ N_{2}\\ N_{12}\\ M_{1}\\ M_{2}\\ M_{12} \end{array}\right]=\left[\begin{array}{cccccc} A_{11} & A_{12} & 0 & 0 & 0 & 0\\ & A_{22} & 0 & 0 & 0 & 0\\ & & A_{33} & 0 & 0 & 0\\ & & & D_{11} & D_{12} & 0\\ & \text{sym}. & & & D_{22} & 0\\ & & & & & D_{33} \end{array}\right]\cdot \left[\begin{array}{c} \varepsilon_{1}\\ \varepsilon_{2}\\ \varepsilon_{12}\\ \kappa_{1}\\ \kappa_{2}\\ 2\cdot \kappa_{12} \end{array}\right]. \end{array}$$

**Figure 1:**
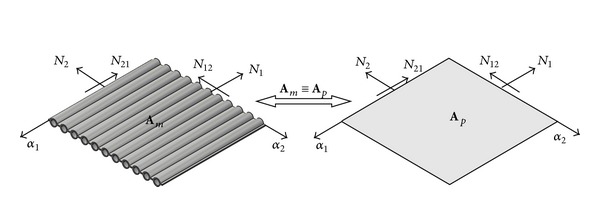
Section forces in the membrane wall, that is, the equivalent orthotropic plate, with the membrane stiffness of membrane wall **A**_*m*_ equivalent to the membrane stiffness of orthotropic plate **A**_*p*_.

**Figure 2:**
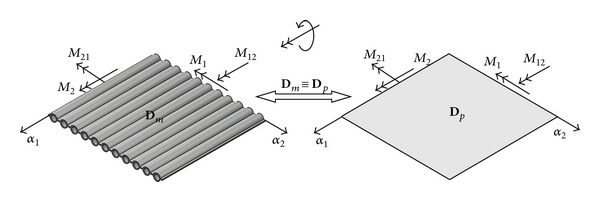
Section moments in the membrane wall, that is, the equivalent orthotropic plate, with the bending stiffness of membrane wall **D**_*m*_ equivalent to the bending stiffness of orthotropic plate **D**_*p*_.

*N*_1_, *N*_2_, and *N*_12_ are the section forces (Figure 1), and, *M*_1_, *M*_2_ and *M*_12_ are the section moments (Figure 2) in the membrane wall, that is, the equivalent orthotropic plate. *A*_11_, *A*_12_, *A*_22_, and *A*_33_ are the members of elasticity matrix for membrane loads, and *D*_11_, *D*_12_, *D*_22_, and *D*_33_ are the members of elasticity matrix for bending loads of equivalent orthotropic plate. *ε*_1_, *ε*_2_, and *ε*_12_ are the membrane deformations, and *κ*_1_, *κ*_2 _, and *κ*_12_ are the curvatures of neutral surface of structurally orthotropic plate, that is, the equivalent orthotropic plate.

**Figure 3:**
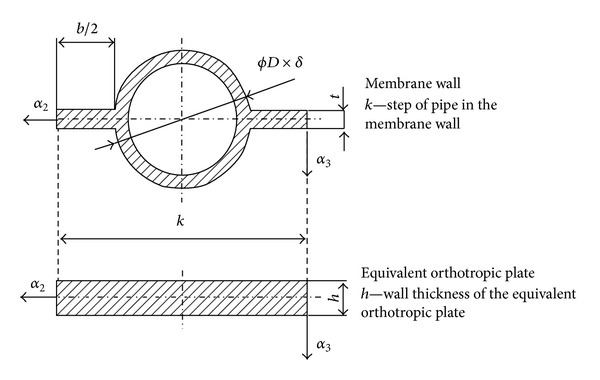
Geometry of cross-section of membrane wall and equivalent orthotropic plate.

The members of elasticity matrix referring to the membrane stiffness [30, 34] of membrane wall can be calculated using the following expressions:

(3) A11=Em,1·h1−νm,12·νm,21,  A12=Em,2·h·νm,121−νm,12·νm,21,A22=Em,2·h1−νm,12·νm,21,  A33=Gm,12·h,

where *E*_*m*,1_ is modulus of elasticity for the membrane stiffness in the direction of axis *α*_1_ (the axis parallel to the axis of pipe in the membrane wall), *E*_*m*,2_ is modulus of elasticity for the membrane stiffness in the direction of axis *α*_2_ (the axis perpendicular to the axis of pipe in the membrane wall), *ν*_*m*,12_ is Poisson's ratio for the membrane stiffness in the direction of axis *α*_1_, *ν*_*m*,21_ is Poisson's ratio for the membrane stiffness in the direction of axis *α*_2_, and *G*_*m*,12_ is shear modulus for the membrane stiffness. The members of elasticity matrix referring to the bending stiffness [29, 33] of membrane wall can be calculated using the following expressions:

(4) D11Eb,1·h312·(1−νb,12·νb,21),D12=Eb,2·h3·νb,1212·(1−νb,12·νb,21),D22Eb,2·h312·(1−νb,12·νb,21),D33=Gb,12·h312,

where the elasticity constants for the bending stiffness are indicated by index *b*. The expression for the wall thickness of equivalent orthotropic plate [30] can be obtained from the equality of moment of inertia of the cross-section of membrane wall, considering the plane perpendicular to the longitudinal axes of the membrane pipes (Figure 3), and the cross-section of equivalent orthotropic plate. Consider

(5) $$\begin{array}{c} h=\sqrt{\frac{3\cdot \pi }{16\cdot D\cdot k}\cdot \left(D^{4}-{\left(D-2\cdot \delta \right)}^{4}\right)+\frac{\left(k-D\right)\cdot t^{3}}{D\cdot k}}. \end{array}$$

The elasticity constants of equivalent orthotropic plate, for the membrane stiffness, can be calculated using the analytical expressions suggested in the paper [31, 32]. These expressions are obtained by equalizing the deformations of membrane wall and equivalent plate (thickness *h*), with the equivalent membrane load

(6) $$\begin{array}{c} E_{m,1}=\frac{E}{k\cdot h}\left[\frac{\pi }{4}\cdot \left(D^{2}-{\left(D-2\cdot \delta \right)}^{2}\right)+\left(k-D\right)\cdot t\right],\\ E_{m,2}=\frac{k\cdot E}{h\cdot \left[3\cdot {\left(D/\delta -1\right)}^{3}\cdot \left(\pi /8-1/\pi \right)+b/t\right]},\\ G_{m,12}\approx \frac{\sqrt{E_{m,1}\cdot E_{m,2}}}{2\cdot \left(1+\sqrt{\nu_{m,12}\cdot \nu_{m,21}}\right)},\\ \nu_{m,21}=\nu_{m,12}\cdot \frac{E_{m,2}}{E_{m,1}}, \end{array}$$

where *E* is modulus of elasticity, and *ν*_*m*,12_ = *ν* is Poisson's factor for the isotropic material of membrane wall. The expressions for the elasticity constants of equivalent orthotropic plate and for the bending stiffness of membrane wall are proposed in the paper [31, 32]. Same as for the membrane stiffness, these expressions are also obtained by equalizing the deformations of membrane wall and equivalent plate (thickness *h*), with the equivalent bending load

(7) Eb,1=Ek·h3·[3·π16·(D4−(D−2·δ)4)+(k−D)·t3],Eb,2=E·(th)3·(n·k)4∑i=1n(i·k)4−(i·k−b)4,Gb,12≈Eb,1·Eb,22·(1+νb,12·νb,21),νb,21=νb,12·Eb,2Eb,1,

where *n* is number of pipes in the membrane wall and *ν*_*b*,12_ = *ν* is Poisson's factor for the isotropic material of membrane wall. After the homogenization of steam boiler membrane wall, the stiffness matrix of shell element can be calculated according to the expression

(8) $$\begin{array}{c} k=\overset{}{\underset{A}{\int }}B^{T}DB\;dA, \end{array}$$

where *A* is surface of shell element and **B** is operator of boundary magnitude.

## 3. Calculation of Reactions in the Steam Boiler Supports for a Real Model of Membrane Wall Geometry, for the Membrane Walls Approximated by the Equivalent Orthotropic Plate, and for the Ideal Rigid Model of Boiler Geometry

**Figure 4:**
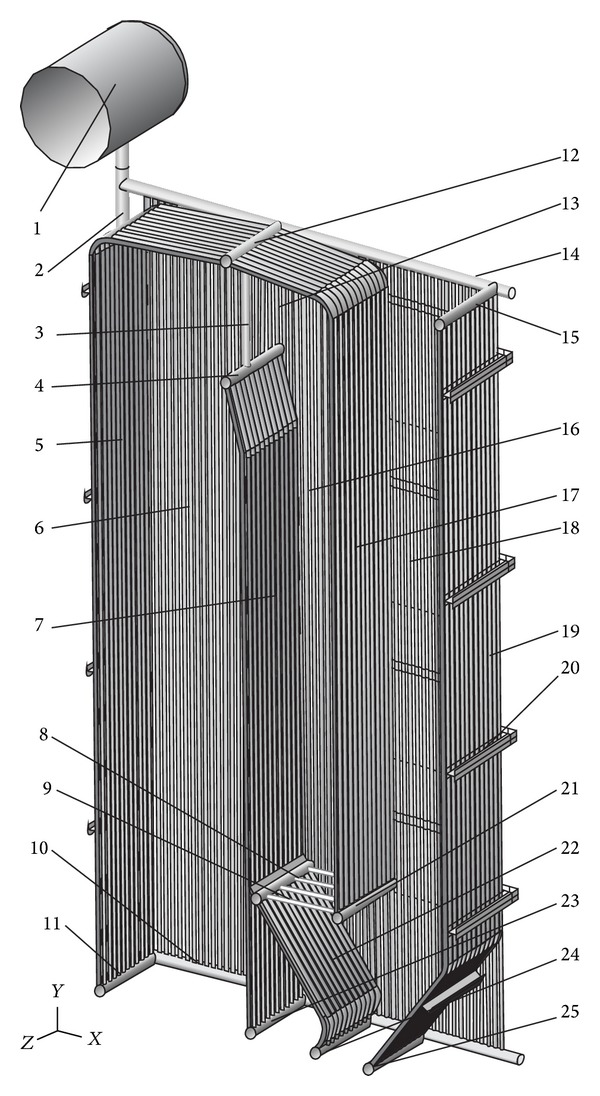
Real calculation geometry model.

**Figure 5:**
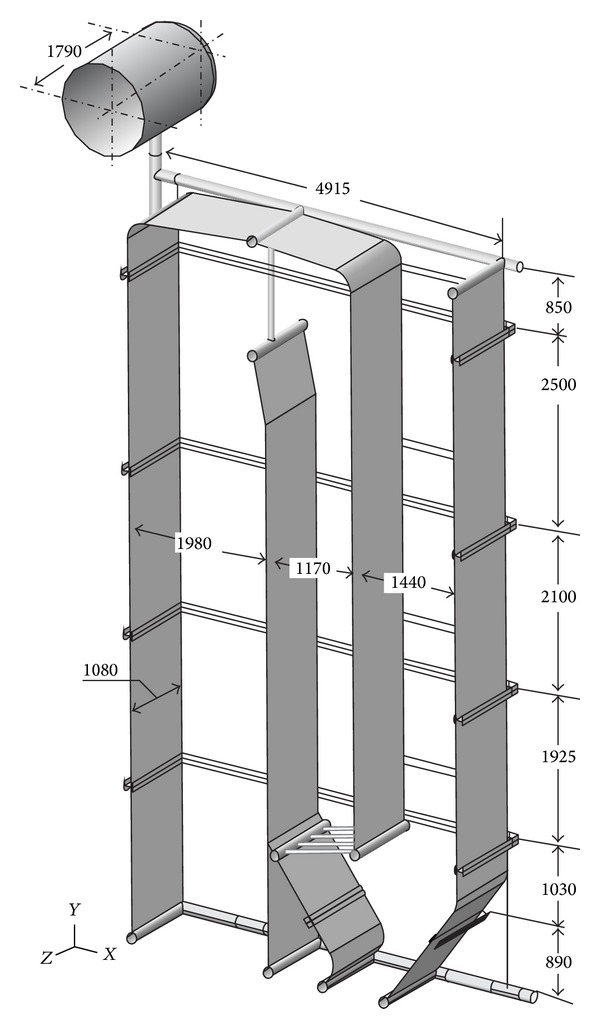
Calculation geometry model approximated as the equivalent orthotropic plate (dimensions are in mm).

The finite element method using Abaqus 6.9-3 will be applied for the calculation of reactions in the supports. The real geometry of membrane walls (Figure 4) and the membrane walls approximated as the equivalent orthotropic plate (Figure 5) will be discretized by a four-node doubly curved thin shell element using the reduced integration with hourglass control. The finite element has the codename S4R5 and it is applied for small deformations and has five degrees of freedom per node.

**Figure 6:**
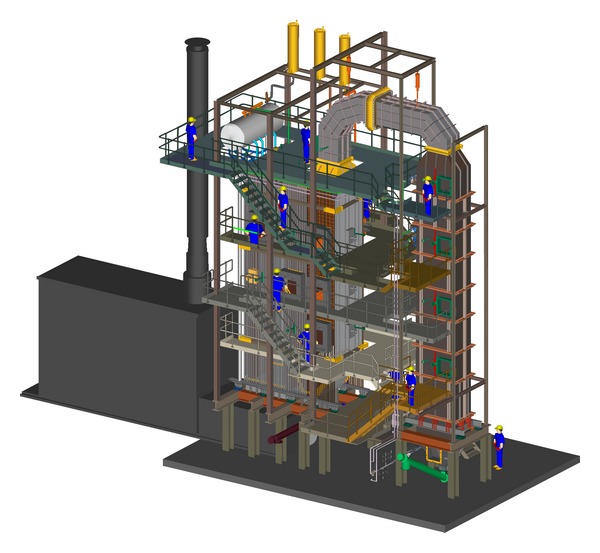
3D-disposition of biomass incineration power plant “Milano.”

The load of the mass of boiler structure will be taken into consideration for the calculation of reactions in the supports of “Milano” steam boiler (Figure 6). Other loads, such as the mass of working fluid, the fouling mass that is deposited on the heating surfaces during the combustion process, the mass of refractory, and the mass of insulation, will not be considered here. Pipe material of membrane walls is P235GH, material of membrane tape and buckstays is S235JRG2, and material of chambers is 16Mo3. All three steels belong to the group of low-carbon steels with ≤0.3% carbon. Since the boiler membrane walls are evaporator heating surfaces, the calculation temperature is *ϑ* = 275°C. Assuming that the mechanical properties of boiler structure are isotropic, [36] the modulus of elasticity for all three materials is approximately equal and amounts to *E* = 187.12 GPa. For the value of Poisson's factor, *ν* = 0.3 is used.

**Figure 7:**
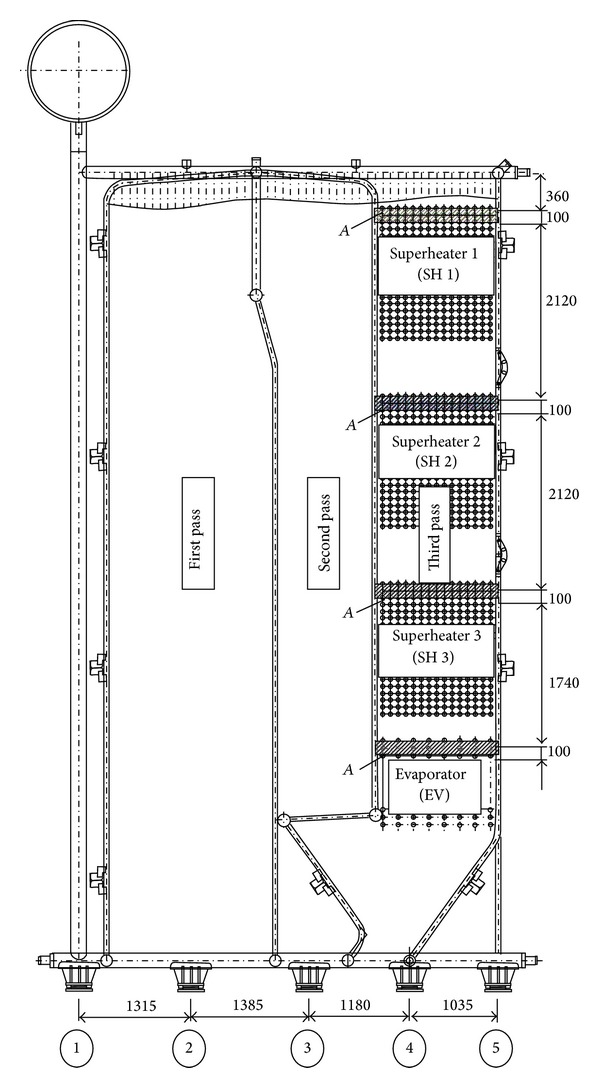
Position of boiler supports and heat exchangers (dimensions are in mm).

Due to the single boiler symmetry, for the calculation using the finite element method, it is sufficient to model only one-half of the boiler geometry (Figures 4 and 5). The boiler is supported by ten supports, five on the distributor chamber of the left side wall and five on the distributor chamber of the right side wall (Figure 7). In the third pass of the boiler, the heat exchangers were installed, that is, one evaporator and three superheaters. The heat exchangers were supported on the side walls of the third pass. Due to the weight of heat exchangers, the load of the left and right side wall of the third pass is identical. For the calculation of reactions in the boiler supports, half of the weight of individual heat exchanger will be used as the equivalent tangential surface pressure on the side wall of the third pass. This pressure is applied on surface A of membrane wall on which the carriers of heat exchangers are welded (Figure 7). Bending of the side membrane walls of the third pass, due to the load of heat exchangers, was not taken into consideration.

The masses of individual structural elements of boiler, marked according to Figures 4 and 7 and Table 1, are given in Table 2. The inhomogeneity of material of boiler structure will not be taken into consideration here. It is assumed that the geometry of boiler is ideal, without the tolerances of production and assembly, as well as the tolerances of semifinished rolled products built into the boiler. It is assumed that the mechanical properties of material of boiler structure are isotropic, that is, that anisotropy of the welded joints and semifinished rolled products does not influence significantly the reactions in the boiler supports.

The mass of half of boiler without the heat exchangers is *m*′ = 10671 kg, and with the heat exchangers it is *m* = 13621 kg. The membrane walls of “Milano” boiler are made with the step of 90 mm. Two dimensions of the cross-section of pipe (*ϕ*57 × 4 mm and *ϕ*48.3 × 4.5 mm) are applied, as well as the membrane tape (thickness, 6 mm). For the boiler membrane walls, it is possible to calculate the elasticity constants of equivalent orthotropic plate (Table 3) according to expressions (6) and (7). The members of elasticity matrix (Tables 4 and 5), which will be applied to the finite element of equivalent orthotropic plate, will be calculated according to expressions (3) and (4).

In this paper, the results of six different calculations of reactions in the supports of “Milano” boiler will be presented: the calculation of real geometry of boiler (REAL) discretized by the shell finite element, the calculation with the approximation of membrane walls as the plate/shell of equivalent stiffness (APPROX), and the calculation where the boiler is considered as a rigid body (RIGID). Each calculation is made with the load of the mass of boiler, with and without the mass of heat exchangers.

**Figure 8:**
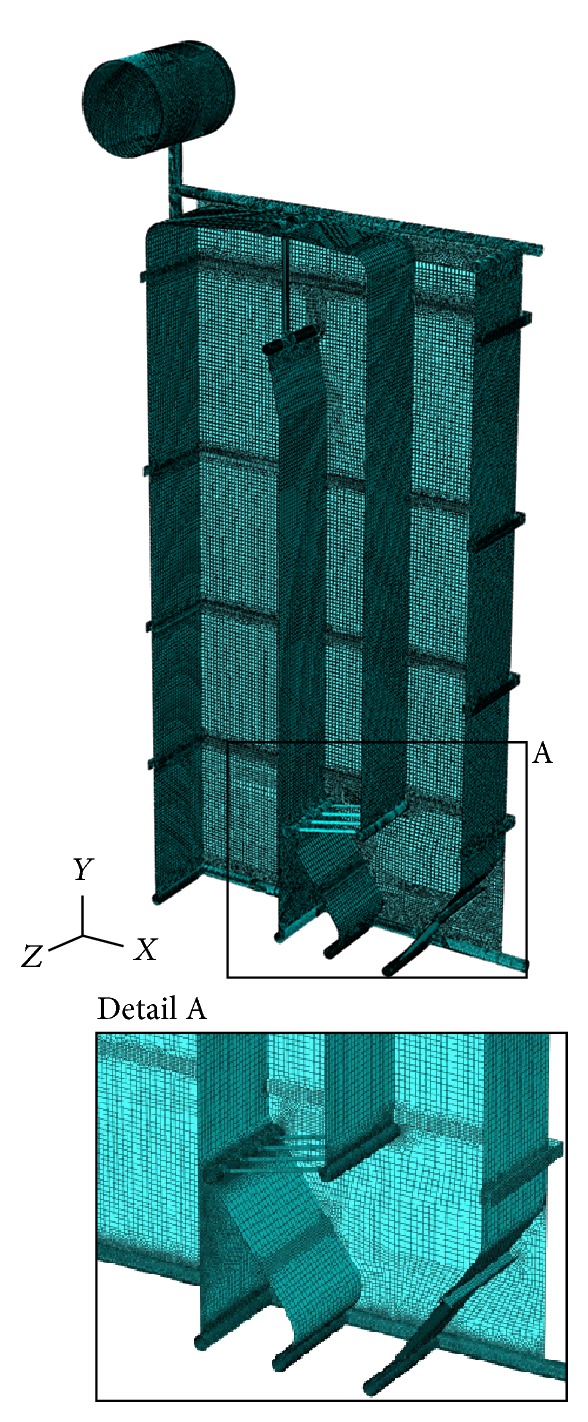
Finite element mesh-calculation model APPROX.

**Figure 9:**
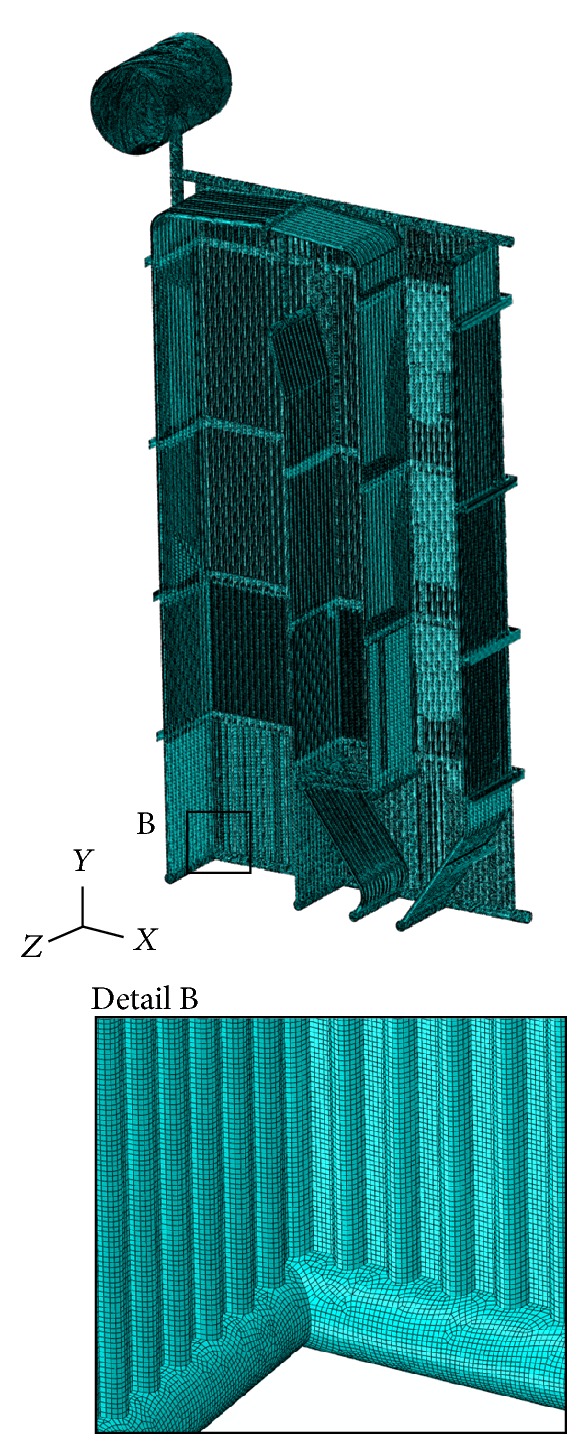
Finite element mesh-calculation model REAL.

The calculation model for the real geometry of boiler consisted of 18943590 variables that include the degrees of freedom and Lagrange multipliers, and for the approximated geometry of boiler membrane walls as the plate/shell of equivalent stiffness, the calculation model consisted of 1619526 variables (degrees of freedom and Lagrange multipliers). In both cases, 4-node finite element S4R5 was predominantly used, and an extremely triangular element was created by the collapse of one node of 4-node element S4R5 (Figures 8 and 9).

The reaction forces in the supports for the influence of total mass of boiler are given in Table 6, and the reaction forces for the influence of mass of boiler without the mass of heat exchangers are given in Table 7. The reaction forces in the boiler supports are denoted according to Figure 7. In Tables 6 and 7, the deviations of reaction forces for calculation APPROX (compared to calculation REAL) were calculated, and the deviation was expressed as a percentage and marked as Δ_*A*_. In the same way, the deviation of reaction forces for calculation RIGID (compared to calculation REAL) was calculated, and it was expressed as a percentage and marked as Δ_*K*_. The deviations were calculated according to the following expressions:

(9) $$\begin{array}{c} \Delta_{A}=\left(\frac{F_{\text{Ri}}\left(\text{APPROX}\right)}{F_{\text{Ri}}\left(\text{REAL}\right)}-1\right)\cdot 100,\%,\\ \Delta_{K}=\left(\frac{F_{\text{Ri}}\left(\text{RIGID}\right)}{F_{\text{Ri}}\left(\text{REAL}\right)}-1\right)\cdot 100,\%. \end{array}$$

## 4. Conclusion

The investigation of the influence of approximation of steam boiler membrane walls, using the equivalent orthotropic plate for the calculation of reactions in the boiler supports, is presented in this paper. The expressions for the elasticity constants that have been published in previous papers [30–32] were applied.

- The reactions in the boiler supports were calculated with the assumption that the structure of boiler is ideally rigid.
- The calculations were carried out for the load of the mass of boiler, with and without the mass of heat exchangers in the third pass of boiler.
- The number of variables for the calculation model REAL was more than 11 times higher than for the calculation model APPROX. Therefore, the calculation of node values of model REAL lasted for 1 h, 23 min and 50 s, and for the model APPROX 1 min and 56 s. The calculation was carried out on a PC with CPU Intel(R) Core (TM) i7, 24 GB RAM, and 64-bit operating system.
- The deviations of results for the reactions in the supports for the calculation APPROX, compared to the calculation REAL, are max. 6.31% for the load of the total mass of boiler and 6.01% for the load without the mass of heat exchangers.
- The deviations of results for the calculation RIGID, compared to the calculation REAL, are 78.75% for the load without the mass of heat exchangers.
- If the results of calculation REAL are accepted as approximately accurate, then it can be concluded that the application of calculation model APPROX is acceptable for practical use. Due to the huge error, the calculation model RIGID is not recommended for practical use.
- Using the method of equivalent stiffness of membrane wall, more accurate results of calculation could be acquired if, when calculating the modulus of elasticity *E*_*b*,2_, the membrane pipe would be observed as a solid body, and not as an ideal rigid body [32].
- Additional improvements could be achieved by taking into consideration the transverse shear stiffness.

## Figures and Tables

**Table 1:** List of positions according to Figure 4.

Pos.	Type	Dimensions	Pos.	Type	Dimensions
1	Shell and end of drum	*ϕ*1200 × 35 mm	14	Collector header of side wall	*ϕ*139.7 × 12.5 mm

2	Downcomers	*ϕ*168.3 × 16 mm	15	Collector header of rear wall of third pass	*ϕ*139.7 × 12.5 mm

3	Connecting pipe	*ϕ*88.9 × 8.8 mm	16	Side wall of second pass	Pipe: *ϕ*57 × 4 mm Tape: *t* = 6 mm Step: *k* = 90 mm Number of pipes: *n* = 12

4	Collector header of rear wall of first pass	*ϕ*139.7 × 12.5 mm	17	Rear wall of second pass	Pipe: *ϕ*57 × 4 mm Tape: *t* = 6 mm Step: *k* = 90 mm Number of pipes: *n* = 23

5	Front wall of first pass	Pipe: *ϕ*57 × 4 mm Tape: *t* = 6 mm Step: *k* = 90 mm Number of pipes: *n* = 23	18	Side wall of third pass	Pipe: *ϕ*48.3 × 4.5 mm Tape: *t* = 6 mm Step: *k* = 90 mm Number of pipes: *n* = 15

6	Side wall of first pass	Pipe: *ϕ*57 × 4 mm Tape: *t* = 6 mm Step: *k* = 90 mm Number of pipes: *n* = 21	19	Rear wall of third pass	Pipe: *ϕ*57 × 4 mm Tape: *t* = 6 mm Step: *k* = 90 mm Number of pipes: *n* = 23

7	Rear wall of first pass	Pipe: *ϕ*57 × 4 mm Tape: *t* = 6 mm Step: *k* = 90 mm Number of pipes: *n* = 23	20	Buckstays	HE-B120

8	Collector header of wall of hopper	*ϕ*139.7 × 12.5 mm	21	Distributor header of rear wall of second pass	*ϕ*139.7 × 12.5 mm

9	Connecting pipes	*ϕ*48.3 × 4 mm	22	Wall of hopper	Pipe: *ϕ*57 × 4 mm Tape: *t* = 6 mm Step: *k* = 90 mm Number of pipes: *n* = 23

10	Distributor header of side wall	*ϕ*168.3 × 20 mm	23	Distributor header of rear wall of first pass	*ϕ*139.7 × 12.5 mm

11	Distributor header of front wall	*ϕ*139.7 × 12.5 mm	24	Distributor header of wall of hopper	*ϕ*139.7 × 12.5 mm

12	Collector header of front and rear wall of second pass	*ϕ*139.7 × 12.5 mm	25	Distributor header of rear wall of third pass	*ϕ*139.7 × 12.5 mm

13	Side wall in transition zone	Pipe: *ϕ*57 × 4 mm Tape: *t* = 6 mm Step: *k* = 90 mm Number of pipes: *n* = 34			

**Table 2:** Masses of constituent structural elements for half geometry of “Milano” boiler.

Pos.	Mass, kg	Pos.	Mass, kg	Pos.	Mass, kg	Pos.	Mass, kg	Pos.	Mass, kg
1	1967	7	639	13	315	19	786	25	43
2	595	8	43	14	197	20	668	SH1	1185
3	23	9	17	15	43	21	43	SH2	1185
4	43	10	402	16	702	22	157	SH3	527
5	893	11	43	17	721	23	43	EV	53
6	1184	12	43	18	1018	24	43		

**Table 3:** Elasticity constants and wall thickness of equivalent orthotropic plate for membrane walls of “Milano” boiler.

Membrane wall (position)	*h*, mm	*E*_*m*,1_, GPa	*E*_*m*,2_, GPa	*G*_*m*,12_, GPa	ν_*m*,21_	*E*_*b*,1_, GPa	*E*_*b*,2_, GPa	*G*_*b*,12_, GPa	ν_*b*,21_
5, 7, 17, 19, 22	23.48	76.492	1.367	4.915	0.005361	454.159	8.064	29.095	0.005327
6	23.48	76.492	1.367	4.915	0.005361	454.159	8.023	29.025	0.005300
13	23.48	76.492	1.367	4.915	0.005361	454.159	8.203	29.336	0.005419
16	23.48	76.492	1.367	4.915	0.005361	454.159	7.690	28.439	0.005080
18	20.40	88.592	3.880	8.722	0.013138	442.956	9.583	31.199	0.006490

**Table 4:** Members of elasticity matrix of equivalent orthotropic plate for membrane walls of “Milano” boiler for membrane stiffness.

Membrane wall (position)	*A*_11_, N/mm	*A*_12_, N/mm	*A*_22_, N/mm	*A*_33_, N/mm
5, 6, 7, 13, 16, 17, 19, 22	1799282	9646	32152	115438
18	1814749	23843	79476	177966

**Table 5:** Members of elasticity matrix of equivalent orthotropic plate for membrane walls of “Milano” boiler for bending stiffness.

Membrane wall (position)	*D*_11_, N·mm	*D*_12_, N·mm	*D*_22_, N·mm	*D*_33_, N·mm
5, 7, 17, 19, 22	491000497	2615341	8717804	31404889
6	490996567	2602240	8674134	31329272
13	491014120	2660750	8869166	31665404
16	490964096	2494004	8313348	30696524
18	314156438	2038906	6796354	22084218

**Table 6:** Reactions in the boiler supports for the influence of total mass.

Reactions in boiler supports	*F*_*R*1_, N	*F*_*R*2_, N	*F*_*R*3_, N	*F*_*R*4_, N	*F*_*R*5_, N
REAL	22748	27020	32657	38515	12676
APPROX	24183	25992	31015	40026	12499
RIGID	27234	22608	31088	36609	16077
Δ_*A*_, %	6.31	−3.80	−5.03	3.92	−1.40
Δ_*K*_, %	19.72	−16.33	−4.80	−4.95	26.83

**Table 7:** Reactions in the boiler supports for the influence of mass of boiler without the mass of heat exchangers.

Reactions in boiler supports without the mass of heat exchangers	*F*_*R*1_, N	*F*_*R*2_, N	*F*_*R*3_, N	*F*_*R*4_, N	*F*_*R*5_, N
REAL	24329	29007	24633	21190	5521
APPROX	25790	27681	23752	21822	5574
RIGID	27397	21905	34552	10957	9869
Δ_*A*_, %	6.01	−4.57	−3.58	2.98	0.96
Δ_*K*_, %	12.61	−24.48	40.27	−48.29	78.75
